# A Novel Selenium-Based Nanozyme (GSH-Se) Ameliorates Colitis in Mice by Modulating the Nrf2/Keap1 and GPx4 Pathways

**DOI:** 10.3390/ijms26051866

**Published:** 2025-02-21

**Authors:** Caimei Wu, Yuwei Zhang, Ziyun Zhou, Kun Zhang, Yixuan Zhou, Jiayong Tang, Ruinan Zhang, Hua Li, Fali Wu, Shipping Bai, Xianxiang Wang, Yang Lyu

**Affiliations:** 1 Animal Nutrition Institute, Sichuan Agricultural University, Chengdu 611130, China; caimeiwu@sicau.edu.cn (C.W.);; 2 Key Laboratory of Animal Disease-Resistance Nutrition, Sichuan Province, Ministry of Agriculture and Rural Affairs, Ministry of Education, Chengdu 611130, China; 3 College of Science, Sichuan Agricultural University, Chengdu 611130, China

**Keywords:** selenium, glutathione, nanozyme, antioxidants, IBD

## Abstract

Combination of selenium (Se) and glutathione peroxidase (GPx) can reduce the dose of Se used while concurrently exploiting their antioxidative performance, which can be used as a potential treatment for ulcerative colitis. Nanozymes possess higher stability, are more economical, and have more multifunctionalities than natural enzymes and thus could be an ideal approach for their combination. Therefore, this study synthesised a nanozyme using glutathione (GSH) and Se—GSH-Se—and evaluated its alleviating effects on colitis in mice induced by dextran sulphate sodium salt (DSS). Three doses of GSH-Se, 6 mM, 12 mM, and 18 mM were supplemented in DSS-induced colitis in mice. Findings showed that GSH-Se supplementation ameliorated colitis by improving the colonic mucosal integrity, reducing inflammatory responses and oxidative stress, and alleviating gut microbiota imbalance in mice with DSS-induced colitis. Moreover, an in vitro experiment was performed to unravel the molecular mechanism by which GSH-Se ameliorated colitis in mice, based on lipopolysaccharide-induced inflammation in mouse colon epithelial cells. The results suggested that the alleviating effects of GSH-Se on mouse colitis was likely mediated by the activation of the Nrf2/Keap1 (nuclear factor E2-related factor 2/Kelch-like ECH-associated protein 1) and GPx4 signalling pathways.

## 1. Introduction

Ulcerative colitis (UC), a chronic inflammatory bowel disease (IBD), presents as a persistent assault on the colonic mucosa, characterised by recurrent episodes of bloody diarrhoea, abdominal pain, and weight loss [1]. This inflammatory cascade is driven by a complex interplay of genetic predisposition, environmental factors, and an aberrant immune response [1,2]. Common medications used to manage UC include amino-salicylates (e.g., sulfasalazine and mesalamine), corticosteroids (e.g., prednisone and budesonide), immunomodulators (e.g., azathioprine and cyclosporine), and others (e.g., tumour necrosis factor alpha inhibitors) [1,3]. Nevertheless, these medications often fail to provide a cure and are associated with considerable side effects, highlighting the need for the development of novel, safe, and effective therapeutic agents [1,2,3].

Whilst the precise aetiology remains obscure, a growing body of evidence points towards a central role of oxidative stress in perpetuating the disease process [4]. Oxidative stress, a state of imbalance between the production of reactive oxygen species (ROS) and the body’s antioxidant defence mechanisms, emerges as a potent mediator in UC pathogenesis [2,4]. Excessive ROS damage cellular components like lipids, proteins, and DNA, leading to inflammation and tissue damage in the gut [2,3,4,5]. Dysregulated immune responses contribute to the pro-inflammatory milieu in the colon, further escalating the production of ROS [5]. Continuous exposure of the inflamed intestinal mucosa to the damaging effects of ROS overproduction compromises gastrointestinal function—including nutritional malabsorption, increased intestinal permeability, and disturbed gut motility—activates pro-inflammatory signalling pathways, and further exacerbates inflammation [4,5,6]. Specifically, the Nrf2/Keap1 (nuclear factor E2-related factor 2/Kelch-like ECH-associated protein 1) and GPx4 (glutathione peroxidase) signalling pathways activate cellular adaptive responses to various oxidative stress damages, representing key targets in UC pathogenesis [4,5,6].

The role of antioxidants in mitigating the damaging effects of oxidative stress in UC/IBD is gaining significant attention [7]. Antioxidants, substances capable of neutralising ROS, offer a potential therapeutic strategy to counter the progression of UC/IBD [8]. Studies have explored the use of exogenous antioxidants, such as vitamins [9], polyphenols [10], and N-acetylcysteine [11], to reduce oxidative damage and ameliorate symptoms in UC patients. Several studies have also investigated modulators for inflammatory signalling pathways (e.g., aurothioglucose and ebselen as GPx inhibitors) as a potential therapeutic strategy for UC [12,13]. However, the efficacy of antioxidant therapy remains a subject of ongoing investigation. Whilst some studies have demonstrated promising results in reducing inflammation and improving clinical outcomes [7,8], further exploration is essential to delineate the intricate mechanisms by which oxidative stress contributes to UC pathogenesis and to optimise the therapeutic potential of antioxidants.

Selenium (Se), an essential trace mineral, plays a crucial role in maintaining cellular health and mitigating the deleterious effects of oxidative stress [14]. Its unique antioxidant properties are primarily attributed to its incorporation into seleno-proteins [15], a diverse family of proteins with vital functions in cellular redox regulation, by which selenium acts as a potent defender against ROS [16]. In addition, its ability to modulate inflammatory responses, by suppressing the production of pro-inflammatory cytokines, plays a significant role in mitigating the damaging effects of chronic inflammation [14,15,16]. Furthermore, selenium contributes to a robust immune system by supporting the activity of immune cells and reducing oxidative stress within these cells, enhancing their ability to combat diseases [17]. In general, the antioxidant properties of selenium make it clinically significant and valuable in combating UC. However, excessive Se intake can disrupt cellular processes, cause toxicity (i.e., selenosis), and lead to various health issues (e.g., gastrointestinal/nervous system/respiratory problems) [14,15]. The tolerable upper intake for Se for adults is 400 µg/day [14]. Maintaining a balanced approach is therefore essential for reaping the benefits of Se supplementation without risking toxicity [15].

Interestingly, GPx, a key enzyme in the body’s antioxidant defence system [18], relies heavily on selenium for its catalytic activity [19]. This enzyme utilises glutathione (GSH, C_10_H_17_N_3_O_6_S), a tripeptide, as an essential cofactor, which facilitates the efficient reduction of hydrogen peroxide and lipid hydroperoxides, thus preventing ROS formation and protecting cellular components from oxidative damage [18,19]. Thus, integrating the application of Se with GPx offers a compelling avenue for exploration. This dual methodology has the potential to enhance intestinal antioxidative capacity and combat colitis, whilst simultaneously affording a reduction in the required Se concentration and thereby limiting the possibility of adverse effects from excessive exposure.

Nanozymes, nanoscale materials with simulated enzyme activity, present advantages of higher stability, being more economical, and having more multifunctionalities compared to natural enzymes [20]. Due to the flexibility in the composition and structural design [21], nanozymes offer an unparalleled opportunity for combining Se and GPx which could leverage both of their antioxidative performances while simultaneously minimising the risk of Se toxicity. To this end, we synthesised a GSH- and Se-based nanozyme, GSH-Se, which has been demonstrated to present both GPx-like activity and Se-like antioxidative performance in vitro. Nevertheless, it is still unknown whether GSH-Se can relieve colitis, and in vivo mechanisms by which it alleviates oxidative stress remain unclear. Therefore, the present study aimed to evaluate the relieving effect of GSH-Se on colitis in mice, attempting to unravel its mechanism of reducing oxidative damage. The findings of this study could provide a new approach for the treatment of UC/IBD.

## 2. Results

### 2.1. Toxicity of GSH-Se

The results of GSH-Se toxicity are presented in Supplementary Figure S1. There was no significant difference in body weight and aspartate aminotransferase (AST) and alanine aminotransferase (ALT) levels (Figure S1A–D) after 7-day and 30-day 18 mM GSH-Se treatment in mice (*p* > 0.05). No significant difference was observed in cell viability after 20–100 µg/mL of GSH-Se treatment (Figure S1E) (*p* > 0.05).

### 2.2. DSS-Induced Colitis and the Attenuating Effects of GSH-Se

**Figure 1:**
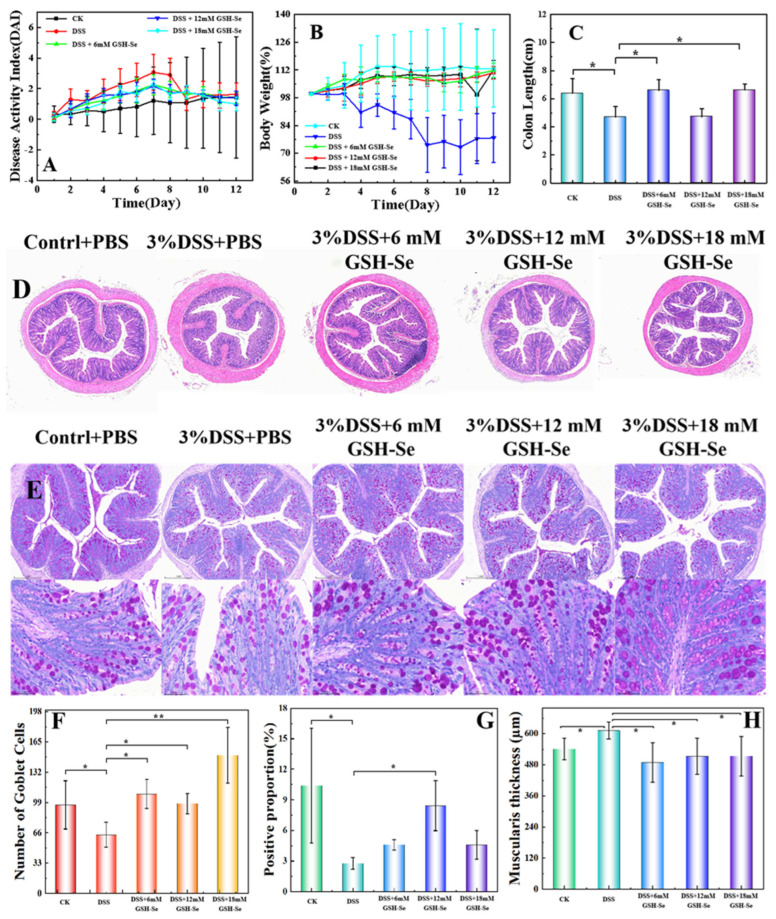
Effects of DSS and GSH-Se on colitis status and colonic morphology in mice. (**A**) DAI index; (**B**) weight loss; (**C**) length of colon; (**D**,**E**) colonic mucosal morphology, scale bar = 20 µm; (**F**) number of colonic mucosal goblet cells; (**G**) proportion of positive mucin MUC2 in colonic mucosa; (**H**) wrinkle thickness of colonic mucosa. CK: control check; DSS: dextran sulphate sodium salt; PBS: phosphate-buffered saline. * Significant difference (*p* < 0.05), ** extremely significant difference (*p* < 0.01) by Tukey’s multiple comparisons; *n* = 10.

Compared to the CK (control check) group, DSS (dextran sulphate sodium salt) treatment resulted in obvious damage to colonic mucosal morphology (i.e., an increase in wrinkle thickness and crypt depth, Figure 1D, and a reduction in goblet cells, Figure 1E), an increase in the disease activity index (DAI) score (Figure 1A) and a decrease in daily body weight (Figure 1B), and a significant reduction in colon length (Figure 1C), number of goblet cells (Figure 1F), proportion of Mucin 2 (MUC2; Figure 1G), and wrinkle thickness of the colonic mucosa (Figure 1H) (*p* < 0.05).

Compared to the DSS group, GSH-Se supplementation apparently ameliorated colonic morphological injury (i.e., a decrease in wrinkle thickness and crypt depth, Figure 1D, and an increase in goblet cells, Figure 1E) and returned the reduced DAI score (Figure 1A) and daily body weight (Figure 1B) to levels closer to the CK group. Specifically, the supplementation of 6 mM and 18 mM GSH-Se increased the length of the colon (Figure 1C), the number of global cells (Figure 1F), and wrinkle thickness of colonic mucosa (Figure 1H) (*p* < 0.05); in addition to the increase in these indexes, the 12 mM GSH-Se supplementation also increased the MUC2 proportion of colonic mucosa (Figure 1G) (*p* < 0.05).

### 2.3. Effects of DSS and GSH-Se on Oxidative Status

The results of the effects of DSS and GSH-Se on oxidative status are shown in Table 1. Compared to the CK group, DSS treatment significantly decreased total superoxide dismutase (T-SOD) activities and GSH levels in the colon and serum, decreased total antioxidant capacity (T-AOC) activities in the serum, and decreased GPx activities and increased malondialdehyde (MDA) levels in the three samples (*p* < 0.05). Compared to the DSS group, the addition of 6 mM GSH-Se significantly increased GPx activities and GSH levels in the colon and serum and decreased MDA levels in the serum (*p* < 0.05); the addition of 12 mM GSH-Se significantly increased the activities of GPx and T-SOD and the levels of GSH in the colon and serum and decreased MDA levels in the liver and serum (*p* < 0.05); the addition of 18 mM GSH-Se significantly increased GPx activities and GSH levels and decreased MDA levels in the three samples, as well as increasing T-AOC activities in the serum and T-SOD activities in the colon and serum (*p* < 0.05).

### 2.4. Effects of DSS, LPS, and GSH-Se on Inflammatory Responses

Table 2 presented data on the effects of DSS, LPS (lipopolysaccharide), and GSH-Se on inflammatory responses in mice and MCEC cells (colonocytes of mouse colon epithelial cells). For the in vivo study, DSS treatment significantly increased the levels of interleukin 1 beta (IL-1β), interleukin 6 (IL-6), tumour necrosis factor gamma (TNF-γ), and tumour necrosis factor alpha (TNF-α) compared to the CK group (*p* < 0.05). Compared to the DSS group, the supplementation of 6 mM GSH-Se significantly decreased the levels of IL-1β and IL-6 (*p* < 0.05); the supplementation of 12 mM GSH-Se significantly decreased the levels of IL-6, TNF-γ, and TNF-α (*p* < 0.05); and the supplementation of 18 mM GSH-Se significantly decreased the levels of IL-1β and IL-6 (*p* < 0.05).

For the in vitro study, LPS treatment significantly increased the levels of IL-1β, IL-6, and TNF-α compared to the CK group, while 18 mM GSH-Se supplementation significantly decreased the levels of IL-1β and IL-6 compared to the LPS group (*p* < 0.05).

### 2.5. Effects of DSS and GSH-Se on the Colonic Microbiome

**Figure 2:**
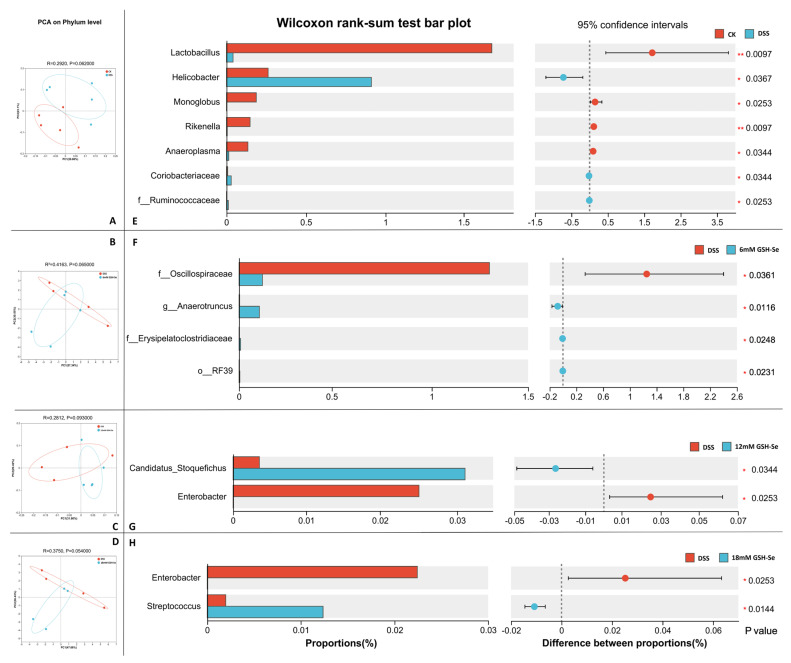
Effects of DSS and GSH-Se on the colonic microbiome (*n* = 10). (**A**–**D**) Principal component analysis at the phylum level in CK vs. DSS, DSS vs. DSS + 6 mM GSH-Se, DSS vs. DSS +12 mM GSH-Se, and DSS vs. DSS +18 mM GSH-Se group, respectively (*p* < 0.10); (**E**–**H**) differential bacteria at the genus level in CK vs. DSS, DSS vs. DSS + 6 mM GSH-Se, DSS vs. DSS + 12 mM GSH-Se, and DSS + DSS vs. 18 mM GSH-Se group, respectively, * *p* < 0.05, ** *p* < 0.01.

At the phylum level, a different colonic microbial community was observed in CK vs. DSS (r = 0.292, *p* = 0.062; Figure 2A), DSS vs. 6 mM GSH-Se (r = 0.416, *p* = 0.065; Figure 2B), DSS vs. 12 mM GSH-Se (r = 0.281, *p* = 0.093; Figure 2C), and DSS vs. 18mM GSH-Se (r = 0.375, *p* = 0.054; Figure 2D), respectively.

Specifically, several significantly differential bacterial genera were observed. Compared to the CK group, DSS treatment significantly increased the abundance of f_Ruminococcaceae (an unknown genus belongs this family, same as below) and the genera *Helicobacter* and *Coriobacteriaceae-UCG* and decreased the abundance of the genera *Lactobacillus*, *Monoglobus*, *Rikenella*, and *Anaeroplasma* (Figure 2E) (*p* < 0.05). Compared to the DSS group, the supplementation of 6 mM GSH-Se significantly decreased the abundance of f_Oscillospiraceae, increased the abundance of o_RF39, f_Erysipelatoclostridiaceae, and the genus *Anaerotruncus* (Figure 2F); the 12 mM GSH-Se supplementation decreased the abundance of the genus *Enterobacter* and increased the abundance of Candidatus_Stoquefichus (Figure 2G); the 18 mM GSH-Se supplementation decreased the abundance of the genus *Enterobacter* and increased the abundance of the genus *Streptococcus* (Figure 2H) (*p* < 0.05).

**Figure 3:**
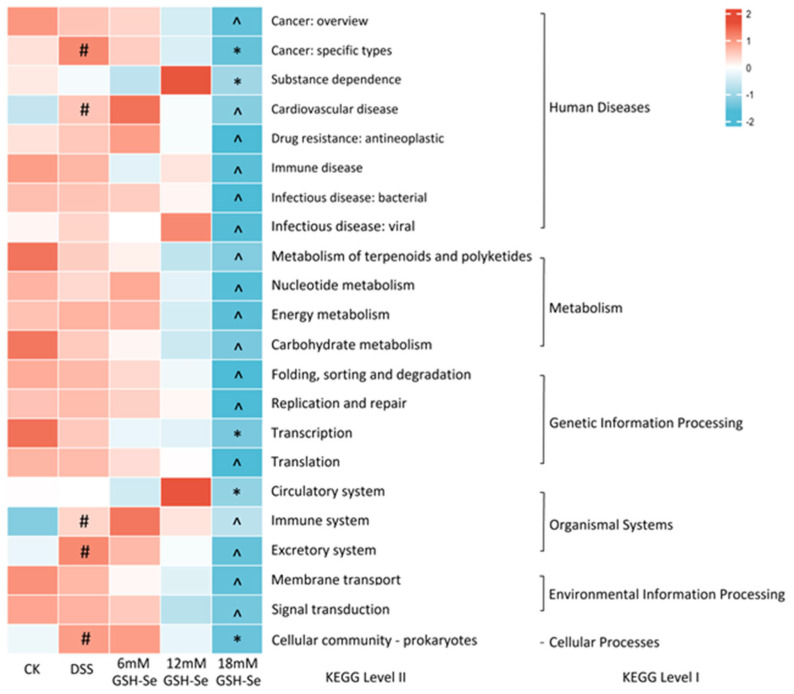
Functional prediction analysis based on level I and level II KEGG pathways. Blocks with colours in the heatmap represent log_10_ transferred relative abundance of pathways. # means a significant difference between the CK and DSS group (*p* < 0.05), * means a significant difference between the DSS and DSS + 18 mMGSH-Se group (*p* < 0.05), and ^ means a changing trend between the DSS and DSS + 18 mM GSH-Se group (0.05 < *p* < 0.10) by Tukey’s multiple comparisons; *n* = 10.

Further functional prediction by pathway analysis is presented in Figure 3. Compared to the CK group, DSS treatment significantly increased the abundance of five pathways including cancer (specific types), cardiovascular disease, the immune system, the excretory system, and the cellular community—prokaryotes (*p* < 0.05). Compared to the DSS group, the 18 mM GSH-Se supplementation decreased the abundance of five pathways including cancer (specific types), substance dependence, transcription, the circulatory system, and the cellular community—prokaryotes (*p* < 0.05). In addition, all other pathways in the 18 mM GSH-Se group showed decreasing trends compared to the DSS group (*p* < 0.10).

### 2.6. Effect of DSS, LPS, and GSH-Se on the Nrf 2/Keap 1/GPx 4 Pathway

**Figure 4:**
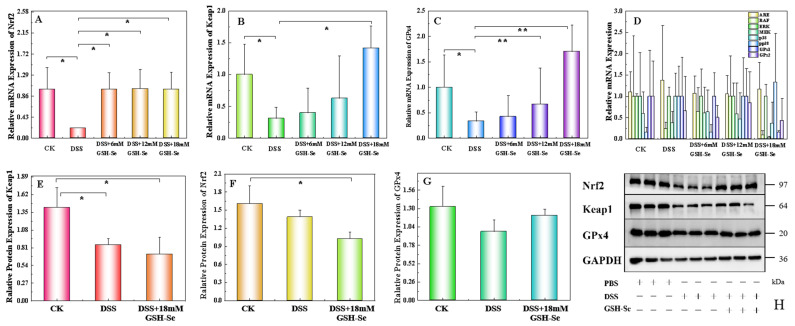
Expression levels of mRNA and protein in the Nrf2/Keap1/GPx4 pathway in the colon of mice (*n* = 10), * *p* < 0.05, ** *p* < 0.01. (**A**) Nrf2 (nuclear factor E2-related factor 2) mRNA expression; (**B**) Keap1 (Kelch-like ECH-associated protein 1) mRNA expression; (**C**) Gpx4 (glutathione peroxidase 4) mRNA expression; (**D**) expression of oxidative and inflammatory-related mRNA, including ARE (AU-rich element), RAF (serine/threonine kinase-1), ERK (extracellular signal-regulated kinase), MEK (mitogen-activated protein), p38 (mitogen-activated protein kinases 38 kDa protein), pp38 (38 kDa phosphoprotein), GPx1, and GPx2; (**E**) Keap1 protein expression; (**F**) Nrf2 protein expression; (**G**) Gpx4 protein expression; and (**H**) western blotting for Nrf2 (97 kDa), Keap1 (64 kDa), and Gpx4 (20 kDa) protein. The loading control is set as GAPDH (glyceraldehyde-3-phosphate dehydrogenase, 36 kDa).

Results of the mRNA and protein expression in mice are presented in Figure 4. Compared to the CK group, DSS treatment significantly decreased the expression levels of Nrf2, Keap1, and GPx4 mRNA and Keap1 protein (*p* < 0.05). Compared to the DSS group, three concentrations of GSH-Se significantly increased Nrf2 mRNA expression levels, 12 mM and 18 mM GSH-Se supplementation increased GPx4 mRNA expression levels, and 18 mM GSH-Se also increased Keap1 mRNA expression levels (*p* < 0.05). No significant difference was observed for oxidative and inflammatory-related mRNA (ARE, RAF, ERK, MEK, p38, pp38, GPx1, and GPx2) and protein Nrf2 and GPx4 (*p* > 0.05).

**Figure 5:**
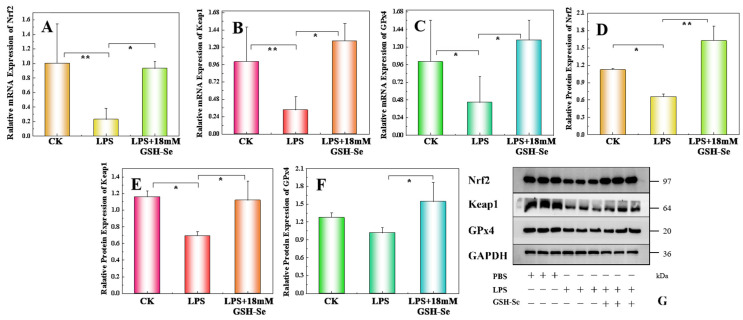
Expression levels of mRNA and protein in the Nrf2/Keap1/GPx4 pathway in MCEC cells (*n* = 6), * *p* < 0.05, ** *p* < 0.01. (**A**) Nrf2 mRNA expression; (**B**) Keap1 mRNA expression; (**C**) Gpx4 mRNA expression; (**D**) Nrf2 protein expression; (**E**) Keap1 protein expression; (**F**) Gpx4 protein expression; and (**G**) western blotting for Nrf2 (97 kDa), Keap1 (64 kDa), and Gpx4 (20 kDa) protein. The loading control is set as GAPDH (36 kDa).

Results of the in vitro study are shown in Figure 5. Compared to the CK group, LPS treatment significantly decreased the expression levels of Nrf2, Keap1, and GPx4 mRNA and Keap1 and Nrf2 proteins (*p* < 0.05). Compared to the LPS group, GSH-Se supplementation significantly increased the expression levels of Nrf2, Keap1, and GPx4 mRNA and these proteins (*p* < 0.05).

## 3. Discussion

Nanozymes present a compelling alternative to conventional enzymes because of their high stability and biocompatibility; nanozymes with antioxidative properties can thus replace traditional enzymes for treating UC/IBD [20,21]. Furthermore, due to its tunability, combing nanozymes with another potential antioxidant can enhance the performance and offer a more promising therapeutic strategy [20,21]. Considering the remarkable antioxidative effects of both GPx and selenium [14,18], a novel nanozyme GSH-Se was synthesised, and its attenuating effect on mouse colitis was evaluated in this study.

Numerous studies have highlighted the antioxidant capacity of selenium, which is mediated through various mechanisms, such as the incorporation into seleno-proteins [14], modulation of inflammatory responses [15], improvement of cellular immune functions [14], and stimulation of GPx activity [17]. Nevertheless, excessive Se intake can also lead to toxicity and organism damage [22], biosafety evaluation of GSH-Se is thereby extremely crucial. Fortunately, all the three levels of GSH-Se did not show any adverse effects in mice (e.g., body weight, AST, and ALT) and MCEC cells (i.e., cell viability), indicating that the GSH-Se used in this study has no observable toxicity to mice and MCEC cells and can be considered safe.

In this study, DSS treatment successfully induced colitis in mice, as evidenced by a significant decrease in body weight, an increase in the DAI score, and morphological damage to the colonic mucosa. More specifically, the DSS treatment resulted in an increase in the wrinkle thickness of the colonic mucosa and significant reductions in colon length, the number of goblet cells, and the proportion of MUC2 secretion in the colonic mucosa. The integrity of the intestinal mucosa is fundamental for ensuring normal intestinal function [23]. Wrinkles in the colonic mucosa play a vital role in gut health by increasing the surface area of the colon [24], thereby enhancing nutrient absorption, mucus production (e.g., MUC2), providing a suitable environment for the microbiome, and supporting robust immune function of colonic cells [25]. Similarly, a healthy colon typically has a sufficient number of goblet cells producing adequate amounts of MUC2, resulting in a strong mucosal barrier and a balanced gut microbiome [26]. Alterations in wrinkle thickness, goblet cell number, and MUC2 secretion can be associated with various gut disorders including IBD. The decreased wrinkle thickness, coupled with the reduced goblet cells and MUC2 secretion observed following DSS induction, suggests that the mucosa thickens in response to inflammation, leading to damage of colonic immune function and mucosal integrity. In contrast, GSH-Se supplementation effectively repaired the mucosal injury caused by DSS treatment, indicated by the recovered body weight, DAI score, and mucosal integrity. These findings demonstrate that GSH-Se supplementation ameliorated DSS-induced colitis in mice.

More specifically, GSH-Se supplementation alleviated DSS-induced colitis by decreasing inflammatory responses and improving the immune system. This is reflected by the recovered levels of four cytokines in the colon (i.e., IL-1β, IL-6, TNF-γ, and TNF-α), which are associated with inflammation and immunomodulation [27]. IL-1β and TNF-α are notably powerful pro-inflammatory cytokines that play pivotal roles in the inflammatory response to tissue damage [28,29]. While IL-6 and TNF-γ are major immunoregulatory cytokines that influence the development of both innate and adaptive immune responses, the surge and accumulation of them can also contribute to inflammation and various diseases, including IBD [30,31]. In general, the addition of GSH-Se reduced the increased levels of these four cytokines caused by DSS-induced colitis, indicating that GSH-Se supplementation can ameliorate inflammatory responses and enhance immune function in colitis in mice.

It is not surprising that the addition of GSH-Se increased GPx activities and GSH levels, as it is synthesised by using GSH as a substrate, and GPx activities are catalysed by Se [19]. In addition, GSH-Se supplementation ameliorated oxidative stress caused by DSS-induced colitis in mice, as indicated by the restoration of reduced total antioxidant capacity (i.e., T-AOC) and T-SOD activities, and the lowering of elevated MDA levels in colitis in mice. It is well known that SOD is a crucial enzyme that protects cells from the damaging effects of superoxide radicals, thereby contributing to overall cellular health and reducing the risk of various diseases [32]. In contrast, MDA levels are often used as a marker of oxidative stress, and its formation contributes to cell membrane disruption, compromised cellular function, and various disease processes [33]. These results suggested that GSH-Se supplementation ameliorated oxidative stress caused by DSS-induced colitis in mice. Specifically, 18 mM appears to be the most efficacious dosage in counteracting oxidative stress, as evidenced by the significant alteration of all five measured parameters. Further research into the dose–response effects of GSH-Se is warranted.

In this study, DSS-induced colitis caused disorders in the gut microbiota. This is demonstrated by the distinct bacterial community (i.e., Figure 2A, clear separation on the principal component analysis (PCA)), and several differential bacterial genera (i.e., increased f_Ruminococcaceae, *Helicobacter*, and *Coriobacteriaceae-UCG*; decreased *Lactobacillus*, *Monoglobus*, *Rikenella*, and *Anaeroplasma*). Studies have shown that *Coriobacteriaceae-UCG* and certain Ruminococcaceae species (e.g., *R. bromii*) are linked to the exacerbation of IBD symptoms [34,35]. While *Helicobacter* is not a direct cause of IBD, it is a common cause of gastritis and peptic ulcers and has been associated with gut dysbiosis and systemic inflammation [36], which may be a consequence of UC development and could potentially influence IBD pathogenesis [37]. In contrast, the four bacteria decreased by UC are generally considered beneficial bacteria in humans and animals alike. *Lactobacillus* species have been frequently used as probiotics due to their beneficial roles in treating various gut disorders (e.g., diarrhoea) and promoting optimal health (e.g., immune modulation) [38]. *Monoglobus* and *Rikenella* can produce short-chain fatty acids (SCFAs), improve gut barrier integrity, and potentially modulate immune responses [39,40]. Although its role in the human gut is not fully understood, a few studies have suggested that *Anaeroplasma* can be used as a potential anti-inflammatory probiotic for the treatment of chronic intestinal inflammation [41]. In general, an increase in the abundance of several inflammatory-related bacteria and a decrease in several beneficial bacteria were observed in colitis in mice, indicating a severe imbalance of gut microbiota by DSS treatment. This is further inflected by the pathway analysis, as the DSS group is associated with five increased pathways related to health disorders, including cancer, cardiovascular disease, the immune system, the excretory system, and the cellular community.

Conversely, GSH-Se supplementation showed a positively regulatory effect on the microbiome imbalance induced by colitis. Clear separations were observed on the PCA plots between the DSS group and three GSH-Se groups (Figure 2B–D), indicating distinct bacterial communities between mice with colitis and mice supplemented with GSH-Se. Moreover, several differential bacteria were associated with GSH-Se supplementation in this study, including decreased f_Oscillospiraceae and *Enterobacter* and increased o_RF39, f_Erysipelatoclostridiaceae, *Anaerotruncus*, and *Streptococcus*. Although few Oscillospiraceae members are capable of producing SCFAs, some studies also observed the potential role of Oscillospireaceae in various gut disorders, including IBD and irritable bowel syndrome [42]. Similarly, despite their capacity of vitamin synthesis, *Enterobacter* as opportunistic pathogens can contribute to inflammation in the gut and potentially exacerbate IBD [43]. In contrast, GSH-Se supplementation also increased four SCFA-producing bacteria that have beneficial potentials. The genera *Anaerotruncus* and *Streptococcus*, as well as many members of the RF39 order (e.g., *Ruminococcus* and *Faecalibacterium*) and the Erysipelatoclostridiaceae family (e.g., *Solobacterium* and *Holdemania*), can break down complex carbohydrates and produce SCFAs (e.g., butyrate), which have beneficial effects on gut barrier integrity and immune modulation [44,45,46,47]. Additionally, research is exploring the potential of *Streptococcus* as probiotics to promote gut health and reduce inflammation [48]. These results indicated that GSH-Se supplementation improved the gut environment in mice with colitis, potentially alleviating the gut dysbiosis caused by DSS-induced colitis. This is also suggested by the functional prediction between the DSS and 18 mM GSH-Se group; 18 mM GSH-Se supplementation decreased five pathways associated with diseases and cell processes (i.e., cancer, substance dependence, transcription, the circulatory system, and the cellular community). Interestingly, pathway analysis did not reveal any significant changes in the 6 mM and 12 mM GSH-Se groups, implying that the 18 mM dose of GSH-Se may have a more pronounced regulatory impact.

To further elucidate the molecular mechanisms underlying the beneficial effects of GSH-Se in alleviating mouse colitis, the expression levels of inflammatory-related mRNA and proteins were determined in this study, and an in vitro study was also conducted using LPS-induced inflammation in MCEC cells. Findings in the present study suggest that the restorative effects of GSH-Se on the colonic barrier integrity and inflammatory responses were likely mediated through the activation of the Nrf2/Keap1 and GPx4 signalling pathways. This was supported by the up-regulated expression levels of Nrf2, Keap1, and GPx4 mRNA and proteins in both mice with colitis and LPS-induced MCEC cells supplemented with GSH-Se. This finding aligns with a recent study that utilised cycloheximide acid as an antioxidant to combat DSS-induced colitis in mice, where colitis was similarly ameliorated by regulating the Nrf2/Keap1 pathway and antioxidative enzymes [49]. Furthermore, comparable results have been observed under other experimental conditions, where selenium-containing nutraceuticals alleviated oxidative stress via Nrf2 and HIF-1α in rats subjected to ischemia–reperfusion [50]. In fact, in the oxidative defence mechanism, the Nrf2/Keap1 signalling pathway activates the cellular adaptive responses in response to a variety of oxidative stress injuries [51]. Specifically, Nrf2 is a key transcription factor regulating oxidative stress [52], and Keap1 protein is a repressor of Nrf2 [53]. Their interaction plays a critical role in various physiological processes, including protection against oxidative stress and inflammation, detoxification of xenobiotics, regulation of cellular growth and development, immune response modulation, and maintenance of tissue homeostasis [54,55]. Furthermore, Nrf2 is a crucial upstream transcription factor that regulates the expression of GPx4 [56]. GPx4 utilises GSH as a reducing substrate to catalyse the detoxification of H_2_O_2_ and lipid hydroperoxides, playing a critical role in antioxidant defence mechanisms by protecting cells from oxidative damage and maintaining membrane integrity [57]. In general, the up-regulation of the Nrf2/Keap1 and GPx4 signalling pathways may be one of the pivotal mechanisms by which GSH-Se alleviates DSS-induced colitis. Consistent with the findings regarding oxidative status and microbiome composition, 18 mM appears to represent the optimal dose for ameliorating DSS-induced colitis. This is evidenced by the most substantial increase observed in the expression of Keap1 and GPx4 proteins in mice (approximately fourfold, Figure 4).

In summary, the supplementation of GSH-Se dramatically ameliorated DSS-induced colitis in mice, substantiated by the increased body weight, DAI score, and mucosal integrity (i.e., decreased wrinkle thickness and crypt depth, increased colon length, goblet cell counts, and MUC2 secretion), the decreased oxidative stress (i.e., increased T-AOC, T-SOD, and GPx activities and GSH levels; decreased MDA levels) and inflammatory responses (i.e., decreased levels of IL-1β, IL-6, TNF-γ, and TNF-α), and the alleviated microbiome dysbiosis (i.e., increased beneficial bacteria, decreased inflammatory-related bacteria and disease-related pathways). Further mechanism analysis revealed that these ameliorating effects were likely mediated by the up-regulation of the Nrf2/Keap1 and GPx4 signalling pathways. All three dosages demonstrated therapeutic benefits without any indications of potential toxicity. Notably, the 18 mM GSH-Se dose demonstrably exerted the most substantial influence, with the majority of the assessed parameters showing the greatest statistically significant changes. The findings suggest that the observed effects may be dose-dependent, with the greatest efficacy occurring at the highest concentration tested.

This study also includes some limitations. For instance, the used dosage and time were relatively limited, the minimal effective dose and the long-term effects as well as side effects were not determined. This study also lacks comparison with the effects of other antioxidants. The protein expression results, with the exception of Keap1, did not reach statistical significance. Further evidence is required to substantiate the involvement of the Nrf2/Keap1/GPx4 pathway. Moreover, the potential of GSH-Se to target the MAPK-NF-κB/AP-1 signalling pathway [58] as well as HIF-1α [50,59] was not investigated in the present study and warrants consideration. Further exploration is imperative to ascertain the optimal dosage and to identify the maximal dosage that yields therapeutic efficacy without any potential adverse effects. Future investigations should encompass the long-term and dose–response effects of GSH-Se, alongside more in-depth mechanistic studies and comparisons with alternative antioxidant-based therapies. Finally, the beneficial effects of GSH-Se offer a promising avenue for combination therapies with other potential strategies to mitigate UC and/or other health risks, such as plant-based high-fibre diets, regular physical activity, and/or diverse nutraceuticals and/or antioxidant/anti-inflammatory compounds [60].

## 4. Materials and Methods

### 4.1. Synthesis of GSH-Se

GSH-Se was synthesised by a hydrothermal method using GSH and Na_2_SeO_3_ as previously described [61]. Briefly, 0.014 g GSH and 0.05 g Na_2_SeO_3_ (Jinshan Chemical Reagent Co., Ltd., Chengdu, China) were mixed with 20 mL of water and placed in a Teflon-lined stainless-steel autoclave. The autoclave was then placed in an oven and reacted at 180 °C for 3 h. After the reaction, the resulting solution was centrifuged at 2248× *g* for 5 min, the supernatant was removed, and it was dialysed for 12 h. The 5-thio-2-nitrobenzoic acid (DTNB, Macklin reagent Co., Shanghai, China) was used as the chromogenic agent to assess the GSH-Px activity of GSH-Se. To this end, 1.9 mL of phosphate-buffered saline (PBS, Sigma-Aldrich, St. Louis, MO, USA), along with 25 µL of GSH, DTNB, GSH-Se, and H_2_O_2_, were successively added to a 2 mL centrifuge tube. The mixture was then reacted in a 37 °C water bath for 15 min, with the ratio exhibiting the highest enzyme activity selected as the optimal synthesis ratio. The results showed that the enzyme activity reached its optimum under the conditions of 37 °C, pH 8, and 25 min [61]. Multiple approaches including X-ray photoelectron spectroscopy (ESCALAB 250Xi, Thermo Fisher, Waltham, MA, USA), Fourier transform infrared spectrometer (Equinox 55, Bruker Optics, Ettlingen, Germany), transmission electron microscope (JEOL 2010, Tokyo, Japan), and X-ray powder diffraction were employed to characterise the morphology, structure, and elemental composition of the prepared GSH-Se. The results indicated that the GSH-Se nanozyme is a spherical aggregate with an average particle size of 350 nm [61].

### 4.2. Animal and Experimental Design

#### 4.2.1. The Use of Animals and Cells

Seventy specific pathogen-free grade C57/BL6 mice (male, 18–20 g, 6 weeks old) were purchased from Dasuo Experimental Animal Technology (Co., Ltd., Chengdu, China). The mice were housed at a controlled room temperature (22 ± 2 °C) and humidity (40–60%) with a 12 h light/dark cycle. The mice were provided with free access to water and food for a week prior to the trial for adaptation. Dextran sulphate sodium salt (DSS, Sigma-Aldrich, St. Louis, MO, USA) was used to induce colitis in the mice, with PBS (Sigma-Aldrich, St. Louis, MO, USA) serving as a control. The animal study was conducted according to the Animal Research: Reporting of in vivo Experiments guidelines, reviewed and approved by the Institutional Animal Care and Use Committee of Sichuan Agricultural University (Chengdu, China, approval code: 20230115W; issue date: 05-January-2023; validity period: January 2023–December 2024).

MCEC colonocytes were obtained from the American Type Culture Collection (ATCC, Manassas, VA, USA) and utilised for in vitro experiments. The cells were cultured in Dulbecco’s modified complete media (DMEM-F12 + 10% FBS + 1% S/P, no pyruvate) in cell culture flasks at 37 °C and 5% CO_2_ in a humidified incubator. The medium was refreshed every two days until the cells reached approximately 80% confluence. The cells were then digested with trypsin, and the number of viable cells was determined using a cell counting plate. The cell concentration was adjusted, and the cells were seeded into cell culture plates. LPS was used to induce inflammation, with PBS serving as a control. All materials for in vitro experiments were purchased from Sigma-Aldrich (St. Louis, MO, USA).

#### 4.2.2. Evaluation of GSH-Se Biosafety

A preliminary experiment was conducted to evaluate the toxicity of GSH-Se prior to the main trial. Twenty male C57BL/6 mice were randomly divided into four groups: the CK 7d, the GSH-Se 7d, the CK 30d, and the GSH-Se 30d group, with five mice in each group. Mice in the GSH-Se groups were orally administered 200 µL of 18 mM GSH-Se daily. For the CK 7d and GSH-Se 7d groups, body weight was recorded daily, and curves depicting body weight changes were plotted. For the CK 30d and GSH-Se 30d groups, body weight was measured on days 0, 5, 10, 15, 20, 25, and 30, and curves showing body weight changes were plotted. On days 7 and 30, the mice were euthanised, and serum samples were collected for analysis of AST and ALT levels.

For the in vitro evaluation, MCEC cells were cultured until they reached 80% confluence. The cells were then treated with GSH-Se at concentrations of 0, 20, 40, 60, 80, and 100 µg/mL for 12 h. The dosages were selected based on results of our preliminary experiment, in which 100 µg/mL was the potentially highest non-toxic concertation [61]. After co-incubation with cell-counting-kit-8 for 2 h, cell viability was assessed.

### 4.3. Experimental Design

A total of 50 mice were included in a single-factor experimental design and randomly assigned to five groups: the CK group, the DSS group, the DSS + 6 mM GSH-Se group, the DSS + 12 mM GSH-Se group, and the DSS + 18 mM GSH-Se group. Mice in the DSS groups received drinking water containing 3% DSS for 7 days, with the DSS solution being refreshed every other day. From day 8 to 11, mice in the CK and DSS groups were administered 200 µL of PBS via oral gavage daily, while mice in the GSH-Se groups received 200 µL of 6, 12, and 18 mM GSH-Se, respectively, also via oral gavage daily. On day 12, the mice were euthanised using carbon dioxide, followed by cervical dislocation, and serum, liver, and colon samples were collected according to established procedures [62,63].

The MCEC cells were divided into three groups: the CK group, the LPS group, and the LPS + GSH-Se group, with six replicates in each group. Once the cell confluence reached approximately 80%, the cells in the LPS and LPS + GSH-Se groups were treated with serum-free medium (DMEM-F12 + 1%S/P + 2 µg/mL LPS, no pyruvate) for 12 h. After discarding the medium, the cells were washed with PBS three times and subsequently treated with serum-free medium (DMEM-F12 + 1%S/P + 18 mM GSH-Se, no pyruvate) for 12 h. Following these treatments, the cells were collected for analysis.

### 4.4. Clinical Evaluation

Colitis status was assessed through evaluation of body and faecal characteristics, the morphology of colon tissue, as well as oxidative stress. Body weight was recorded daily, and weight loss was calculated throughout the trial. Faecal morphology was observed, and a faecal occult blood test kit (Jiancheng Biotechnology Co., Ltd., Nanjing, China) was used to determine the presence of faecal occult blood. A DAI score was also calculated using the criteria outlined in Table S1.

Upon collection, fresh colonic mucosal tissues were fixed in 10% formalin for 48 h and then dehydrated and embedded in paraffin. Embedded tissues were sliced into 4 µm sections (RM2016, Leica, Shanghai, China). The sections were then dehydrated, stained with haematoxylin for 5 min, washed with ddH_2_O, and stained with eosin for 2 min. The sections were then dehydrated and mounted with a neutral resin onto slides. Images were captured on a microscope (TS100, Nikon, Tokyo, Japan) with a CCD (DS-U3, Nikon, Tokyo, Japan) using imaging software (NIS-Elements F3.2, Nikon, Tokyo, Japan) for comprehensive assessment of inflammatory cell infiltration, tissue damage, the positive proportion of Mucin 2, and the number of goblet cells.

### 4.5. Assessment of Oxidative Stress and Inflammatory Status

Oxidative stress was evaluated by spectrophotometry using commercial assay kits (Jiancheng Biotechnology Co., Ltd., Nanjing, China). The activities of GPx, T-AOC, and T-SOD and the levels of GSH and MDA were measured in serum, colon, liver, and cell samples. All measurements were strictly followed according to the manufacturer’s instructions.

Inflammatory status was evaluated by enzyme-linked immunosorbent assay using commercial kits (Jiangsu Enzyme Immunoassay Industry Co., Ltd., Yancheng, China), including the measurement of TNF-α, IL-6, IL-8, IL-1β, and TNF-γ. Specific measurement steps were strictly followed in accordance with the manufacturer’s instructions.

### 4.6. Microbiome Analysis

The gut microbiome was analysed by 16S rRNA gene sequencing. Colon content samples were homogenised, and total bacterial DNA was extracted using a commercial kit (Majorbio Technology Co., Ltd., Shanghai, China). DNA size and integrity were assessed by electrophoresis on 1% agarose gels stained with ethidium bromide. DNA concentration and purity were determined by spectrophotometric measurement at 234 nm, 260 nm, and 280 nm. Subsequently, DNA samples were sent to Majorbio Technology for library preparation, sequencing (Illumina MiSeq, San Diego, CA, USA), and bioinformatic analysis (QIIME 2).

### 4.7. mRNA and Protein Expression

The expression levels of inflammatory-related mRNAs were determined using quantitative real-time polymerase chain reaction (qPCR). Total RNA was extracted from mouse colons and cells using TRIzol lysis buffer, followed by reverse transcription. The qPCR reaction was performed using TB Green^TM^ Premix Ex Taq^TM^ II (Novozan, Nanjing, China) according to the manufacturer’s instructions. A CFX96 well real-time fluorescence qPCR instrument was used to detect the expression levels of the target genes. The qPCR results were analysed using the 2^−ΔΔCt^ method. The primer sequences for the target genes and the reference gene are listed in Table S2.

The relative expression levels of the proteins GAPDH, Nrf2, Keap1, and GPx4 were determined by Western blotting using established protocols [62,63]. The antibodies used, Keap1 (A17062, Rabbit pAb, 1:1000), Nrf2 (A0674, Rabbit pAb, 1:1000), GPx4 (A25009, Rabbit mAb, 1:5000), and GAPDH Mouse mAb (AC002, 1:1000), were purchased from ABclonal company (Wuhan, China); horseradish peroxidase-labelled goat anti-mouse immunoglobulin G and goat anti-rabbit immunoglobulin G were purchased from CoWin Biotechnology (Beijing, China).

### 4.8. Statistical Analyses

Microbiome analysis was conducted based on the QIIME 2 platform. PCA was performed to illustrated group differences in the microbial community (β diversity). The Wilcoxon rank-sum test was carried out to compare the differential bacteria species between groups, with *p* values corrected using the false discovery rate.

All remaining data were analysed using one-way analysis of variance in SPSS 28.0. Multiple comparisons between different treatments were performed using the Tukey method. A trend was considered significant at 0.05 ≤ *p* < 0.1, while *p* < 0.05 indicated significant differences. Results are presented as mean ± SEM.

## 5. Conclusions

Overall, the present study synthesised a novel selenium-based nanozyme, GSH-Se, to ameliorate ulcerative colitis in mice. GSH-Se supplementation profoundly alleviated the colonic barrier integrity, inflammatory responses, oxidative stress, and microbiome imbalance in mice with DSS-induced colitis. A further in vitro experiment on the MCEC cells suggested that the restorative effects of GSH-Se may be achieved by the activation of the Nrf2/Keap1 and GPx4 signalling pathways.

## Figures and Tables

**Table 1:** Results of oxidative status in the colon, liver, and serum.

Items	CK	DSS	DSS + 6 mM GSH-Se	DSS + 12 mM GSH-Se	DSS + 18 mM GSH-Se
**Colon**					
GPx (U/mg protein)	129.44 ± 4.57 ^a^	102.01 ± 0.97 ^b^	119.28 ± 3.13 ^ab^	106.83 ± 7.75 ^a^	129.33 ± 9.28 ^a^
T-AOC (U/mg protein)	1.98 ± 0.14	1.86 ± 0.22	1.27 ± 0.10	1.63 ± 0.18	1.45 ± 0.46
T-SOD (U/mg protein)	78.49 ± 1.12 ^a^	70.40 ± 1.27 ^b^	74.85 ± 1.56 ^b^	76.68 ± 1.94 ^a^	76.68 ± 1.94 ^a^
GSH (mg/g protein)	50.23 ± 5.10 ^a^	14.94 ± 1.52 ^c^	26.27 ± 2.99 ^b^	45.93 ± 3.01 ^a^	53.12 ± 3.77 ^a^
MDA (nmol/mL homogenate)	1.45 ± 0.13 ^b^	6.30 ± 0.26 ^a^	5.13 ± 0.54 ^a^	6.40 ± 0.68 ^a^	1.96 ± 0.22 ^b^
**Liver**					
GPx (U/mg protein)	210.41 ± 28.85 ^a^	77.01 ± 14.60 ^b^	83.55 ± 68.09 ^b^	120.99 ± 16.04 ^b^	275.72 ± 12.29 ^a^
T-AOC (U/mg protein)	2.50 ± 0.29 ^b^	4.01 ± 0.24 ^a^	3.01 ± 0.15 ^ab^	3.30 ± 0.35 ^a^	2.61 ± 0.16 ^b^
T-SOD (U/mg protein)	40.68 ± 5.33	42.63 ± 3.23	33.50 ± 2.22	44.02 ± 3.25	44.14 ± 6.52
GSH (mg/g protein)	45.30 ± 7.45 ^b^	46.08 ± 3.59 ^b^	40.54 ± 2.83 ^b^	52.77 ± 7.16 ^b^	97.09 ± 10.24 ^a^
MDA (nmol/mL homogenate)	2.54 ± 0.36 ^b^	5.87 ± 0.79 ^a^	4.13 ± 0.70 ^ab^	3.71 ± 0.41 ^b^	3.74 ± 0.68 ^b^
**Serum**					
GPx (U/mg protein)	455.03 ± 7.98 ^a^	175.94 ± 9.40 ^d^	275.81 ± 5.42 ^b^	243.77 ± 5.74 ^c^	458.71 ± 3.26 ^a^
T-AOC (U/mg protein)	1.33 ± 0.10 ^a^	0.64 ± 0.01 ^c^	0.62 ± 0.01 ^c^	0.71 ± 0.01 ^c^	0.97 ± 0.13 ^b^
T-SOD (U/mg protein)	84.56 ± 1.96 ^b^	68.81 ± 0.69 ^c^	77.68 ± 1.08 ^bc^	285.54 ± 13.94 ^a^	94.71 ± 1.56 ^b^
GSH (mg/g protein)	197.21 ± 5.28 ^a^	173.22 ± 8.88 ^b^	197.62 ± 3.08 ^a^	191.61 ± 4.39 ^a^	210.59 ± 6.13 ^a^
MDA (nmol/mL supernatant)	3.25 ± 0.92 ^bc^	7.54 ± 0.24 ^a^	4.15 ± 0.24 ^b^	2.25 ± 0.35 ^c^	2.48 ± 0.52 ^c^

CK: control check; DSS: dextran sulphate sodium salt; GPx: glutathione peroxidase; T-AOC: total antioxidant capacity; T-SOD: total superoxide dismutase; GSH: glutathione; MDA: malondialdehyde. ^a, b, c, d^ Values with different superscripts in the same row indicate significant statistical differences between them (*p* < 0.05), analysed by Tukey’s multiple comparisons; *n* = 10.

**Table 2:** Results of inflammatory responses in the mouse colon (*n* = 10) and MCEC cells (*n* = 6).

Colon	CK	DSS	DSS + 6 mM GSH-Se	DSS + 12 mM GSH-Se	DSS + 18 mM GSH-Se
IL-1β (ng/L)	181.77 ± 5.40 ^c^	266.58 ± 7.8 ^a^	237.10 ± 8.01 ^b^	164.50 ± 10.04 ^a^	125.51 ± 10.46 ^d^
IL-6 (ng/L)	78.01 ± 9.07 ^b^	107.88 ± 10.56 ^a^	73.82 ± 6.08 ^b^	74.37 ± 1.74 ^b^	72.07 ± 7.10 ^b^
IL-8 (ng/L)	50.99 ± 1.86	54.71 ± 3.60	47.44 ± 2.77	50.97 ± 1.74	53.15 ± 4.56
TNF-γ (pg/mL)	45.75 ± 4.37 ^b^	75.96 ± 1.56 ^a^	67.23 ± 10.36 ^a^	45.36 ± 4.88 ^b^	67.67 ± 7.01 ^a^
TNF-α (pg/mL)	44.33 ± 4.00 ^b^	73.45 ± 1.64 ^a^	65.21 ± 9.91 ^a^	44.24 ± 4.64 ^b^	65.53 ± 6.79 ^a^
**Cell**	**CK**	**LPS**	**LPS + 18 mM GSH-Se**		
IL-1β (ng/L)	136.82 ± 14.53 ^c^	343.12 ± 13.90 ^a^	208.54 ± 9.55 ^b^		
IL-6 (ng/L)	84.47 ± 3.35 ^b^	106.10 ± 5.12 ^a^	88.57 ± 9.78 ^b^		
IL-8 (ng/L)	34.33 ± 1.61	38.09 ± 3.61	46.11 ± 1.25		
TNF-γ (pg/mL)	60.58 ± 3.81	68.15 ± 7.08	63.18 ± 6.81		
TNF-α (pg/mL)	62.59 ± 4.11 ^b^	70.74 ± 7.28 ^a^	65.38 ± 7.06 ^ab^		

CK: control check; DSS: dextran sulphate sodium salt; TNF-α: tumour necrosis factor alpha; IL: interleukin; TNF-γ: tumour necrosis factor gamma; LPS: lipopolysaccharide. ^a, b, c, d^ Values with different superscripts in the same row indicate significant statistical differences between them (*p* < 0.05), analysed by Tukey’s multiple comparisons.

## Data Availability

The data supporting the findings of this study are included within the article. Further inquiries can be directed to the corresponding author.

## Supplementary Materials

The following supporting information can be downloaded at: https://www.mdpi.com/article/10.3390/ijms26051866/s1.

## Abbreviations

The following abbreviations are used in this manuscript:

Se	Selenium
GPx	Glutathione peroxidase
GSH	Glutathione
DSS	Dextran sulphate sodium salt
Nrf2	Nuclear factor E2-related factor 2
Keap1	Kelch-like ECH-associated protein 1
UC	Ulcerative colitis
IBD	Inflammatory bowel disease
ROS	Reactive oxygen species
DNTB	5-thio-2-nitrobenzoic acid
PBS	Phosphate-buffered saline
MCEC	Colonocytes of mouse colon epithelial cells
LPS	Lipopolysaccharide
AST	Aspartate aminotransferase
ALT	Alanine aminotransferase
T-AOC	Total antioxidant capacity
T-SOD	Total superoxide dismutase
MDA	Malondialdehyde
TNF	Tumour necrosis factor
IL	Interleukin
qPCR	Quantitative real-time polymerase chain reaction
PCA	Principal component analysis
SCFAs	Short-chain fatty acids
DAI	Disease activity index

## References

[1] Honap S., Jairath V., Sands B.E., Dulai P.S., Danese S., Peyrin-Biroulet L. (2024). Acute severe ulcerative colitis trials: The past, the present and the future. Gut.

[2] Gros B., Kaplan G.G. (2023). Ulcerative colitis in adults: A review. JAMA.

[3] Honap S., Buisson A., Danese S., Beaugerie L., Peyrin-Biroulet L. (2023). Patient and public involvement in research: Lessons for inflammatory bowel disease. J. Crohn’s Colitis.

[4] Bourgonje A.R., Feelisch M., Faber K.N., Pasch A., Dijkstra G., van Goor H. (2020). Oxidative stress and redox-modulating therapeutics in inflammatory bowel disease. Trends Mol. Med..

[5] Wan Y., Yang L., Jiang S., Qian D., Duan J. (2022). Excessive apoptosis in ulcerative colitis: Crosstalk between apoptosis, ROS, ER stress, and intestinal homeostasis. Inflamm. Bowel Dis..

[6] Hwang J., Jing J., Jeon S., Moon S.H., Park M.Y., Yum D.Y., Kim J.H., Kang J.E., Park M.H., Kim E.J. (2020). SOD1 suppresses pro-inflammatory immune responses by protecting against oxidative stress in colitis. Redox Biol..

[7] Liu Y., Yan H., Yu B., He J., Mao X., Yu J., Zheng P., Huang Z., Luo Y., Luo J. (2022). Protective effects of natural antioxidants on inflammatory bowel disease: Thymol and its pharmacological properties. Antioxidants.

[8] Moura F.A., de Andrade K.Q., Dos Santos J.C.F., Araújo O.R.P., Goulart M.O.F. (2015). Antioxidant therapy for treatment of inflammatory bowel disease: Does it work?. Redox Biol..

[9] Wu Q., Luo Y., Lu H., Xie T., Hu Z., Chu Z., Luo F. (2024). The Potential Role of Vitamin E and the Mechanism in the Prevention and Treatment of Inflammatory Bowel Disease. Foods.

[10] Shapiro H., Singer P., Halpern Z., Bruck R. (2007). Polyphenols in the treatment of inflammatory bowel disease and acute pancreatitis. Gut.

[11] Faghfouri A.H., Zarezadeh M., Tavakoli-Rouzbehani O.M., Radkhah N., Faghfuri E., Kord-Varkaneh H., Tan S.C., Ostadrahimi A. (2020). The effects of N-acetylcysteine on inflammatory and oxidative stress biomarkers: A systematic review and meta-analysis of controlled clinical trials. Eur. J. Pharmacol..

[12] Mertens R.T., Gukathasan S., Arojojoye A.S., Olelewe C., Awuah S.G. (2023). Next generation gold drugs and probes: Chemistry and biomedical applications. Chem. Rev..

[13] Garland M., Hryckowian A.J., Tholen M., Bender K.O., Van Treuren W.W., Loscher S., Sonnenburg J.L., Bogyo M. (2020). The clinical drug ebselen attenuates inflammation and promotes microbiome recovery in mice after antibiotic treatment for CDI. Cell Rep. Med..

[14] Kudva A.K., Shay A.E., Prabhu K.S. (2015). Selenium and inflammatory bowel disease. Am. J. Physiol. Gastrointest. Liver Physiol..

[15] Short S.P., Pilat J.M., Barrett C.W., Reddy V.K., Haberman Y., Hendren J.R., Marsh B.J., Keating C.E., Motley A.K., Hill K.E. (2020). Colonic epithelial-derived selenoprotein P is the source for antioxidant-mediated protection in colitis-associated cancer. Gastroenterology.

[16] Speckmann B., Steinbrenner H. (2014). Selenium and selenoproteins in inflammatory bowel diseases and experimental colitis. Inflamm. Bowel Dis..

[17] Brenneisen P., Steinbrenner H., Sies H. (2005). Selenium, oxidative stress, and health aspects. Mol. Aspects Med..

[18] Flohé L., Toppo S., Orian L. (2022). The glutathione peroxidase family: Discoveries and mechanism. Free Radical Biol. Med..

[19] Handy D.E., Loscalzo J. (2022). The role of glutathione peroxidase-1 in health and disease. Free Radical Biol. Med..

[20] Ai Y., Hu Z.N., Liang X., Sun H.B., Xin H., Liang Q. (2022). Recent advances in nanozymes: From matters to bioapplications. Adv. Funct. Mater..

[21] Zhang R., Yan X., Fan K. (2021). Nanozymes inspired by natural enzymes. Acc. Mater. Res..

[22] Dos Santos M., Penteado J.O., Baisch P.R.M., Soares B.M., Muccillo-Baisch A.L., da Silva Júnior F.M.R. (2021). Selenium dietary intake, urinary excretion, and toxicity symptoms among children from a coal mining area in Brazil. Environ. Geochem. Health.

[23] Breugelmans T., Oosterlinck B., Arras W., Ceuleers H., De Man J., Hold G.L., Hold G.L., De Winter B.Y., Smet A. (2022). The role of mucins in gastrointestinal barrier function during health and disease. Lancet Gastroenterol..

[24] Kai Y. (2022). Mechanical regulation of tissues that reproduces wrinkle patterns of gastrointestinal tracts. Phys. Biol..

[25] Muraleedharan C.K., Mierzwiak J., Feier D., Nusrat A., Quiros M. (2021). Generation of murine primary colon epithelial monolayers from intestinal crypts. J. Vis. Exp..

[26] Yao D., Dai W., Dong M., Dai C., Wu S. (2021). MUC2 and related bacterial factors: Therapeutic targets for ulcerative colitis. eBioMedicine.

[27] Sanmarco L.M., Chao C.C., Wang Y.C., Kenison J.E., Li Z., Rone J.M., Polonio C.M., Gutierrez-Vazquez C., Piester G., Plasencia A. (2022). Identification of environmental factors that promote intestinal inflammation. Nature.

[28] Yu D., Zhao Y., Wang H., Kong D., Jin W., Hu Y., Qin Y., Zhang B., Li X., Hao J. (2021). IL-1β pre-stimulation enhances the therapeutic effects of endometrial regenerative cells on experimental colitis. Stem Cell Res. Ther..

[29] Zhao X., Ma L., Dai L., Zuo D., Li X., Zhu H., Xu F. (2020). TNF-α promotes the malignant transformation of intestinal stem cells through the NF-κB and Wnt/β-catenin signaling pathways. Oncol. Rep..

[30] Jin X., Zimmers T.A., Zhang Z., Pierce R.H., Koniaris L.G. (2010). Interleukin-6 is an important in vivo inhibitor of intestinal epithelial cell death in mice. Gut.

[31] Pott J., Kabat A.M., Maloy K.J. (2018). Intestinal epithelial cell autophagy is required to protect against TNF-induced apoptosis during chronic colitis in mice. Cell Host Microbe.

[32] Islam M.N., Rauf A., Fahad F.I., Emran T.B., Mitra S., Olatunde A., Shariati M.A., Rebezov M., Rengasamy K.R.R., Mubarak M.S. (2022). Superoxide dismutase: An updated review on its health benefits and industrial applications. Crit. Rev. Food Sci. Nutr..

[33] Josh F., Soekamto T.H., Adriani J.R., Jonatan B., Mizuno H., Faruk M. (2021). The combination of stromal vascular fraction cells and platelet-rich plasma reduces malondialdehyde and nitric oxide levels in deep dermal burn injury. J. Inflamm. Res..

[34] Li H., Li H., Stanton C., Ross R.P., Zhao J., Chen W., Yang B. (2024). Exopolysaccharides produced by bifidobacterium longum subsp. Longum ys108r ameliorates dss-induced ulcerative colitis in mice by improving the gut barrier and regulating the gut microbiota. J. Agric. Food Chem..

[35] Willing B.P., Dicksved J., Halfvarson J., Andersson A.F., Lucio M., Zheng Z., Järnerot G., Tysk C., Jansson J.K., Engstrand L. (2010). A pyrosequencing study in twins shows that gastrointestinal microbial profiles vary with inflammatory bowel disease phenotypes. Gastroenterology.

[36] Papamichael K., Konstantopoulos P., Mantzaris G.J. (2014). Helicobacter pylori infection and inflammatory bowel disease: Is there a link?. World J. Gastroenterol..

[37] Koelink P.J., Bloemendaal F.M., Li B., Westera L., Vogels E.W., van Roest M., Gloudemans A.K., van’t Wout A.B., Korf H., Vermeire S. (2020). Anti-TNF therapy in IBD exerts its therapeutic effect through macrophage IL-10 signalling. Gut.

[38] Aghamohammad S., Sepehr A., Miri S.T., Najafi S., Pourshafie M.R., Rohani M. (2022). Anti-inflammatory and immunomodulatory effects of Lactobacillus spp. as a preservative and therapeutic agent for IBD control. Immun. Inflamm. Dis..

[39] Dang J.T., Mocanu V., Park H., Laffin M., Hotte N., Karmali S., Birch D.W., Madsen K.L. (2022). Roux-en-Y gastric bypass and sleeve gastrectomy induce substantial and persistent changes in microbial communities and metabolic pathways. Gut Microbes.

[40] Wu X., Huang X., Ma W., Li M., Wen J., Chen C., Liu L., Nie S. (2023). Bioactive polysaccharides promote gut immunity via different ways. Food Funct..

[41] Khajah M.A., Hawai S. (2023). Effect of minocycline, methyl prednisolone, or combination treatment on the colonic bacterial population in a state of colonic inflammation using the murine dextran sulfate sodium model. Microb. Cell Fact..

[42] Yang J.Y., Chen S.Y., Wu Y.H., Liao Y.L., Yen G.C. (2023). Ameliorative effect of buckwheat polysaccharides on colitis via regulation of the gut microbiota. Int. J. Biol. Macromol..

[43] Lemons J.M., Conrad M., Tanes C., Chen J., Friedman E.S., Roggiani M., Curry D., Chau L., Hecht A.L., Harling L. (2024). Enterobacteriaceae Growth Promotion by Intestinal Acylcarnitines, a Biomarker of Dysbiosis in Inflammatory Bowel Disease. Cell. Mol. Gastroenterol. Hepatol..

[44] Liu B., Wang W., Zhu X., Sun X., Xiao J., Li D., Cui Y., Wang C., Shi Y. (2018). Response of gut microbiota to dietary fiber and metabolic interaction with SCFAs in piglets. Front. Microbiol..

[45] Suzuki I., Shimizu T., Senpuku H. (2018). Role of SCFAs for fimbrillin-dependent biofilm formation of Actinomyces oris. Microorganisms.

[46] Guo X., Cao X., Fang X., Guo A., Li E. (2021). Inhibitory effects of fermented Ougan (Citrus reticulata cv. Suavissima) juice on high-fat diet-induced obesity associated with white adipose tissue browning and gut microbiota modulation in mice. Food Funct..

[47] Ruiz-Saavedra S., Del Rey C.G., Suárez A., Díaz Y., Zapico A., Arboleya S., Salazar N., Gueimonde M., de los Reyes-Gavilán C.G., González S. (2023). Associations of dietary factors and xenobiotic intake with faecal microbiota composition according to the presence of intestinal mucosa damage. Food Funct..

[48] Bailey J.R., Vince V., Williams N.A., Cogan T.A. (2017). Streptococcus thermophilus NCIMB 41856 ameliorates signs of colitis in an animal model of inflammatory bowel disease. Benef. Microbes.

[49] Yang N., Xia Z., Shao N., Li B., Xue L., Peng Y., Zhi F., Yang Y. (2017). Carnosic acid prevents dextran sulfate sodium-induced acute colitis associated with the regulation of the Keap1/Nrf2 pathway. Sci. Rep..

[50] Antonuccio P., Pallio G., Marini H.R., Irrera N., Romeo C., Puzzolo D., Freni J., Santoro G., Pirrotta I., Squadrito F. (2022). Involvement of Hypoxia-Inducible Factor 1-α in Experimental Testicular Ischemia and Reperfusion: Effects of Polydeoxyribonucleotide and Selenium. Int. J. Mol. Sci..

[51] Kansanen E., Kuosmanen S.M., Leinonen H., Levonen A.L. (2013). The Keap1-Nrf2 pathway: Mechanisms of activation and dysregulation in cancer. Redox Biol..

[52] Ma Q. (2013). Role of nrf2 in oxidative stress and toxicity. Annu. Rev. Pharmacol..

[53] Suzuki T., Muramatsu A., Saito R., Iso T., Shibata T., Kuwata K., Kawaguchi S., Iwawaki T., Adachi S., Suda H. (2019). Molecular mechanism of cellular oxidative stress sensing by Keap1. Cell Rep..

[54] Jaramillo M.C., Zhang D.D. (2013). The emerging role of the Nrf2–Keap1 signaling pathway in cancer. Gene. Dev..

[55] Baird L., Dinkova-Kostova A.T. (2011). The cytoprotective role of the Keap1–Nrf2 pathway. Arch. Toxicol..

[56] Shin D., Kim E.H., Lee J., Roh J.L. (2018). Nrf2 inhibition reverses resistance to GPX4 inhibitor-induced ferroptosis in head and neck cancer. Free Radical Biol. Med..

[57] Mayr L., Grabherr F., Schwärzler J., Reitmeier I., Sommer F., Gehmacher T., Niederreiter L., He G., Ruder B., Kunz K.T.R. (2020). Dietary lipids fuel GPX4-restricted enteritis resembling Crohn’s disease. Nature Commun..

[58] Li D., Xie T., Guo T., Hu Z., Li M., Tang Y., Wu Q., Luo F., Lin Q., Wang H. (2023). Sialic acid exerts anti-inflammatory effect through inhibiting MAPK-NF-κB/AP-1 pathway and apoptosis in ulcerative colitis. J. Funct. Foods.

[59] Yin J., Ren Y., Yang K., Wang W., Wang T., Xiao W., Yang H. (2022). The role of hypoxia-inducible factor 1-alpha in inflammatory bowel disease. Cell Biol. Int..

[60] Marini H.R. (2022). Mediterranean Diet and Soy Isoflavones for Integrated Management of the Menopausal Metabolic Syndrome. Nutrients.

[61] Wu C., Zhang Y., Han M., Zhang R., Li H., Wu F., Wu A., Wang X. (2024). Selenium-based nanozyme as a fluorescence-enhanced probe and imaging for chlortetracycline in living cells and foods. Food Chem..

[62] Huang X., He Q., Zhu H., Fang Z., Che L., Lin Y., Xu S., Zhuo Y., Hua L., Wang J. (2022). Hepatic leptin signaling improves hyperglycemia by stimulating MAPK phosphatase-3 protein degradation via STAT3. Cell. Mol. Gastroenterol. Hepatol..

[63] Mou D., Ding D., Yang M., Jiang X., Zhao L., Che L., Fang Z., Xu S., Lin Y., Zhuo Y. (2021). Maternal organic selenium supplementation during gestation improves the antioxidant capacity and reduces the inflammation level in the intestine of offspring through the NF-κB and ERK/Beclin-1 pathways. Food Funct..

