# Supplementary material for: A Novel Selenium-Based Nanozyme (GSH-Se) Ameliorates Colitis in Mice by Modulating the Nrf2/Keap1 and GPx4 Pathways
Source: Int J Mol Sci. 2025 Feb 21;26(5):1866. doi: 10.3390/ijms26051866 (PMC11900211; doi:10.3390/ijms26051866)
Supplement: Supplementary file 1 [file ijms-26-01866-s001.zip › ijms-3474337-supplementary.pdf]

Supplementary Materials

Table. S1 DAI scoring criteria.

Score	Weight change rate(%)	Fecal traits	Fecal latent blood
0	0	Normal	-
1	1-5		+
2	>5-10	Loose stools	++
3	>10-15		+++
4	>15	Diarrhoea	++++

Table. S2 Sequences of the primers for the target genes and the reference genes.

Gene		Primer sequences (5' to 3')	Accessing Number
Keap1	Forward	TGCCCCTGTGGTCAAAGTG	NM_016679
	Reverse	GGTTCGGTTACCGTCCTGC	
NRf2	Forward	GGTCACGCTAATGCAGACAAT	NM_001029878
	Reverse	TCTTCTCAGGGGTATTCGCTTT	
ARE	Forward	TTGAACCAATGGATGGCGTTT	NM_001033426
	Reverse	CTGAATACGCTGATTCTCTGAGC	
RAF	Forward	TTGTCATCGTGGCTCATTACG	NM_134009
	Reverse	CCACTCCCATTTGAGTCTGC	
MEK	Forward	CGAAGAAGAGATGTGGTTCAACA	NM_001160214
	Reverse	TGGTGAGGTTAGTCTTTGTCCC	
ERK	Forward	CATCCCGACTCTCGTTGGAG	NM_001048176
	Reverse	GAATGTGCCAGTGCGTTAGTA	
P38	Forward	CCTATCCTGGAAGAGCCATACT	NM_001004066
	Reverse	ACTTTGTCACGCTGACCAGAT	
P-P38	Forward	GCACCAAAGAGGGGATCTATTC	NM_001013376
	Reverse	ACTTGGCTTCGAGAGTCTGTAA	
GPx1	Forward	GTGCAATCAGTTCGGACACCA	NM_008160
	Reverse	CACCAGGTCGGACGTACTTG	
GPx2	Forward	GCCTCAAGTATGTCCGACCTG	NM_030677
	Reverse	GGAGAACGGGTCATCATAAGGG	
GPx4	Forward	TGTGCATCCCGCGATGATT	NM_008162
	Reverse	CCCTGTACTTATCCAGGCAGA	
β-actin	Forward	ATATTGCTGCGCTCGTGGT	-
	Reverse	TAGGAGTCCTTCTGGCCCAT	

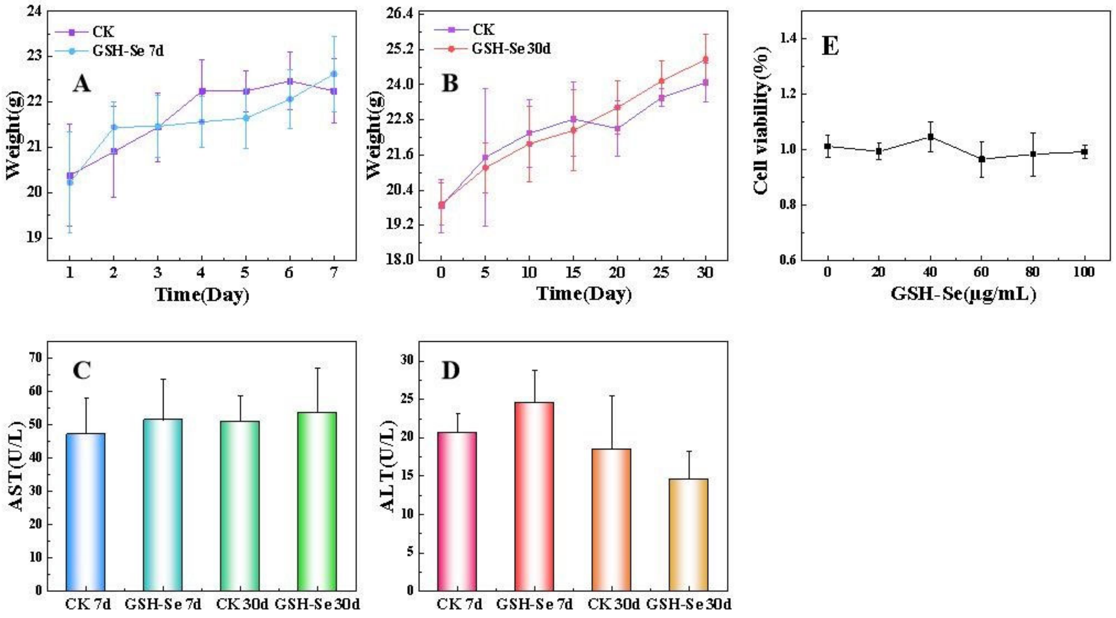

Figure S1. Evaluation of the GSH-Se biosafety. (**A**) weight change in 7 days; (**B**) weight change in 30 days; (**C**) AST levels; (**D**) ALT levels; and (**E**) cell viability.
